# How did COVID-19 lockdown impact the health of older adults in nursing homes? A scoping review

**DOI:** 10.1186/s12877-024-05356-9

**Published:** 2024-09-14

**Authors:** Isabel San Martín-Erice, Paula Escalada-Hernández, Cristina García-Vivar, Sara Furtado-Eraso, Leticia San Martín-Rodríguez, Nelia Soto-Ruiz

**Affiliations:** 1 Department of Health Sciences, Public University of Navarre (UPNA), Avda. de Barañain S/N, Pamplona, Navarra 31008 Spain; 2 IdiSNA, Navarra Institute for Health Research, Pamplona, Spain

**Keywords:** Cognitive state, COVID-19, Emotional state, Functional state, Isolation measures, Lockdown impact, Nursing homes, Older adults, Scoping review

## Abstract

**Background:**

The impact of the COVID-19 pandemic on older adults in nursing homes is significant, with high death rates, disrupted care, isolation measures, and inadequate treatment. Social isolation has increased risks of cognitive disorders, anxiety, and depression. While many studies have examined the pandemic’s effects on nursing home staff and residents’ families, less is known about the health consequences for the residents themselves. This review aims to synthesize literature on the COVID-19 lockdown’s impact on the functional, cognitive, and psycho-emotional states of older adults in nursing homes.

**Methods:**

A scoping review was conducted following the Joanna Briggs Institute guidelines and the PRISMA extension for Scoping Reviews (PRISMA-ScR). Four databases were searched: CINAHL, PubMed, Web of Science, and PsycINFO. The eligibility criteria included studies on older adults in nursing homes during the COVID-19 pandemic, with data that could be disaggregated for this population and results on the lockdown’s impact on physical, cognitive, and psycho-emotional levels.

**Results:**

Seventeen articles met the criteria for data extraction The synthesis was categorized into four main areas: functional, cognitive and psycho-emotional status, and isolation measures. Key findings included decreased functional abilities, lower cognitive test scores during the pandemic’s first waves, development of psychological symptoms, and increased negative feelings among residents.

**Conclusions:**

Highlighting the consequences of confinement for nursing home residents is essential for updating evidence, developing effective strategies, and establishing protocols to mitigate the impact and prevent health issues in future pandemics.

## Background

Since 2020, COVID-19 has affected millions of people around the world, not only due to its impact on health but also on the economy, education, and society in general [1]. The disease has become one of the greatest challenges in global public health, and the population over age 65 has been especially affected by the pandemic. Due to their age and the presence of comorbidities, this population has had a higher risk of developing severe forms of COVID-19 and higher mortality rates than other age groups [2]. In addition, many older adults reside in retirement communities and assisted living facilities, where the pandemic has taken a significant toll.

The alarming number of deaths in nursing homes worldwide underscores the intense suffering experienced by this population, exacerbated by disruptions in their usual care. Thus, frail and disabled nursing homes residents were particularly vulnerable to the virus [3]. Furthermore, nursing homes have organizational characteristics that render them more susceptible to viral spread. First, many residents require professional assistance with dressing, bathing, toileting, and/or ambulation. Second, residents share common activities and spaces in which there is free transit of residents and visitors [4]. For these reasons, as COVID-19 emerged as a health crisis, led to the adoption of recommendations and protocols in nursing homes, as established by international guidelines such as: social distancing and isolation measures, visitors ban, implementing respiratory hygiene protocol, screening for staff and residents, use of personal protective equipment, and instructing staff and residents on appropriate infection prevention procedures [5, 6].

These measures resulted in a significant decrease in infections and deaths within nursing home facilities [7]. In this review, lockdown is defined as: “a temporary condition imposed by governmental authorities (as during the outbreak of an epidemic disease) in which people are required to stay in their homes and refrain from or limit activities outside the home involving public contact (such as dining out or attending large gatherings)” [8].

It has been shown that social isolation increases the risk of cognitive disorders in institutionalized older adults, in addition to causing symptoms of anxiety and depression [9]. It can be assumed that social isolation such as that experienced during the first months of the pandemic has had consequences at the cognitive level, both in people with cognitive deficits and in those not previously diagnosed. Moreover, the functional and psycho-emotional states of older adults living in nursing homes have also been adversely affected. Functional state encompasses the physical ability to perform activities such as self-care, mobility, and independence [10], while cognitive function involves various forms of knowledge and consciousness [11], and psycho-emotional state refers to mental wellbeing and encompasses emotional, psychological, and social aspects [12, 13].

Several scoping reviews have shown the consequences of the COVID-19 pandemic in older adults residing in nursing homes, both physically [14], psychosocially [15], and from a holistic point of view [16] have explored the emotional experiences of not only residents but also family members and caregivers [17]. However, the data from these reviews primarily address the health effects of COVID-19 infection during the first and second waves of the pandemic but there are no reviews encompassing literature that examines the consequences of the implemented lockdown measures. Hence, to comprehensively document the impact of nursing home residents’ confinement across the various waves of the COVID-19 pandemic, it is imperative to update the existing evidence. Additional review is essential to assess the effectiveness of the implemented isolation measures, ultimately enhancing our readiness for future pandemics.

### Objective

The objective of this review is to describe and synthesize scientific literature about the impact of COVID-19 lockdown on the functional, cognitive, and psycho-emotional states of people older than 65 residing in nursing homes.

## Method

### Study design

The scoping review adheres to the Joanna Briggs Institute (JBI) guidelines [18], and complied with Preferred Reporting Items for Systematic Reviews and Meta-Analyses (PRISMA) extension for Scoping Reviews (PRISMA-ScR) for presenting results [19]. The nine steps for conducting a scoping review proposed by the JBI guideline [18] were followed: 1) definition of the aim; 2) development of the inclusion criteria; 3) description of the plan for evidence search and selection, data extraction and presentation of the results; 4) search of the evidence; 5) selection of the evidence; 6) data extraction; 7) analysis of the evidence; 8) presentation of results and 9) summary of the evidence and development of conclusions and identification of the implications of the findings.

### Inclusion and exclusion criteria

The inclusion and exclusion criteria for articles are shown in Table 1.

**Table 1:** Studies eligible criteria

Inclusion criteria	Exclusion criteria
Studies that examined the impact of COVID-19 lockdown on nursing home residents during pandemic	Studies that examined the lockdown impact on older adults’ living at their homes, admitted at acute care units or at intensive care units or older adults attending at day care units
Studies where mixed target groups were included (e.g. health professionals or family members) only admitted if nursing home residents’ data could be disaggregated	Document resources: grey literature (conference proceedings, research reports, memoirs, etc.), letters to the editor, editorials, special articles, commentaries and pilot studies or research protocols
Studies that examined the health impact of COVID-19 lockdown including functional, cognitive, and psycho-emotional results	

### Literature search strategy

A literature review was carried out in the following databases: CINAHL, PubMed, Web of Science and PsycINFO. The search strategy was defined for each of the databases consulted by combining the key terms “elderly, COVID-19, social isolation and nursing home” and related concepts (see Table 2). The terms were combined with the Boolean operators AND and/or OR and truncation (*). The search included studies from January 2020 to August 2024 in English, French, Portuguese and Spanish.

**Table 2:** Search terms (JBI Framework)

POPULATION	CONCEPT	CONTEXT
aged OR elderly OR aged, 80 and over OR oldest old OR geriatrics patients OR older people OR older adults	SARS-CoV-2 OR COVID-19 AND social isolation OR quarantine OR lockdown OR pandemic*	nursing home OR homes for the aged OR long-term care facility* OR care home* OR person institutionalized OR aged care facility OR housing for the elderly OR intermediate care facilities OR skilled nursing facilities OR aged care facilit* OR residential care

* = Truncation

### Study selection

The article selection process was carried out using the Rayyan virtual platform (http://rayyan.qcri.org) for the organization and management of data in systematic and scoping reviews. After eliminating duplicate articles in the different databases, an author performed the first round of screening by reading the titles and abstracts. In the eligibility phase, the full texts were read simultaneously by two authors and were selected following the established selection criteria. Articles lacking consensus underwent review by a third independent reviewer to resolve discrepancies.

### Data extraction and analysis

The data extraction of the selected articles was carried out by two reviewers. An ad hoc template was developed in which the following data were extracted: author, year, country, study objective, data collection period, isolation measure implemented, type of study, sample size and results.

## Results

**Fig. 1:**
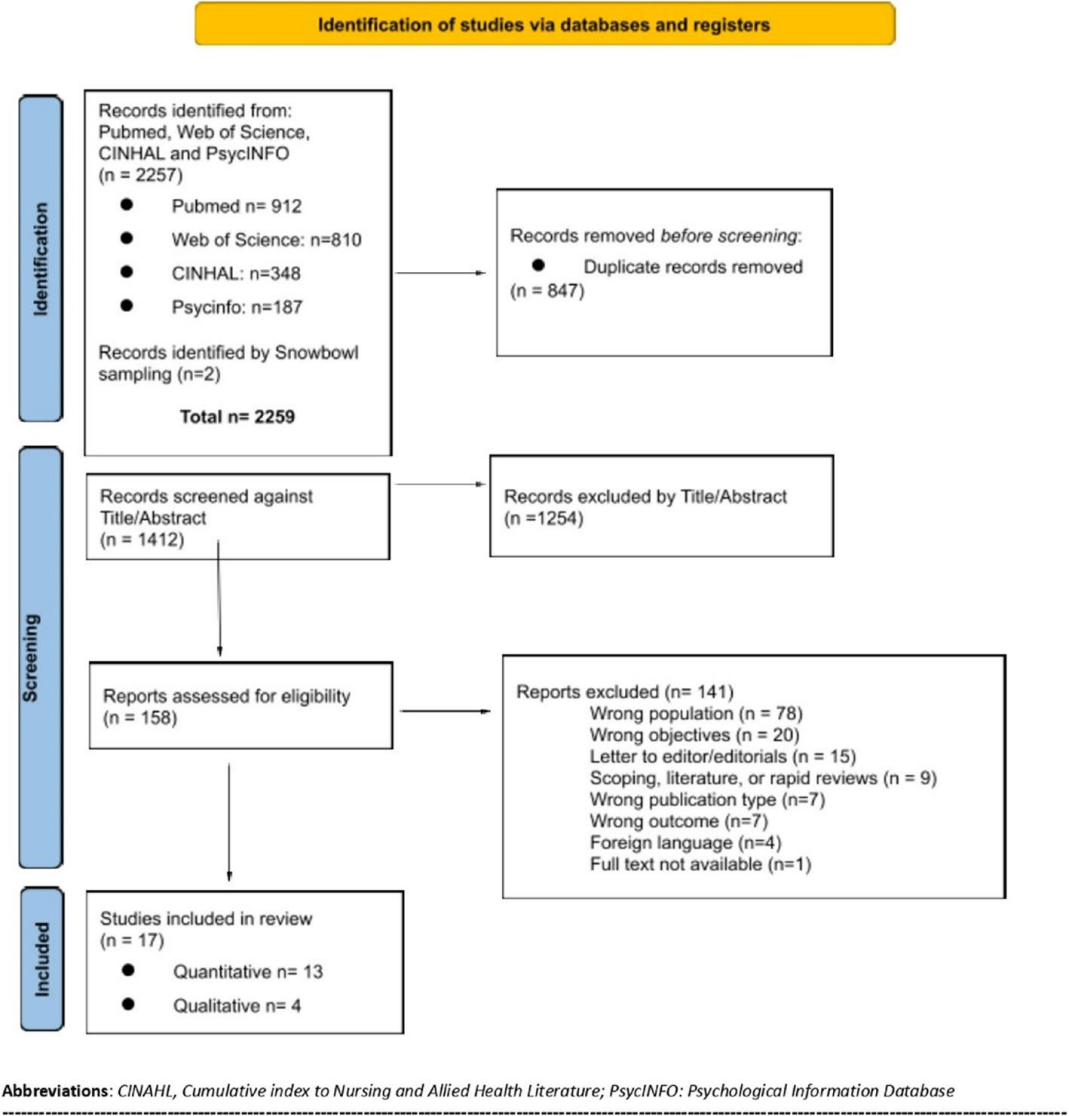
PRISMA-ScR flow diagram

A total of 2259 articles were identified in the database searches. A total of 847 duplicates were eliminated. Of the remaining 1412 articles, 1254 studies were discarded after reading the title and abstract, leaving 158 for a full read. After applying the inclusion criteria, 17 articles met the eligibility criteria and were included for analysis (see Fig. 1). These studies, conducted in Spain (*n* = 3), United States (*n* = 2), France (*n* = 2), Sweden (*n* = 2), Australia (*n* = 1), Poland (*n* = 1), Switzerland (*n* = 1), Belgium (*n* = 1), Malaysia (*n* = 1), Germany (*n* = 1), Canada (*n* = 1) and the Netherlands (*n* = 1), focused on the impact of COVID-19 isolation measures in nursing homes. Their research periods spanned from March 2020 to December 2021. Of the studies that specify their data collection period (*n* = 15), 8 offer data from the initial 3 months of the pandemic, seven from the latter half of 2020, and four from 2021. According to the type of research, there is a majority of quantitative studies (*n* = 13), including longitudinal as well as cross-sectional or retrospective studies. Four studies applied a qualitative approach, applying different analytical methods. All studies were set in nursing homes, evaluating isolation measures, functional, cognitive, and psycho-emotional states of residents.

Next, the general characteristics of these studies are presented along with a synthesis of the results of the studies structured in four main categories based on the identified isolation measures and their impact: (1) isolation measures applied, (2) functional state, (3) cognitive state, and (4) psycho-emotional state (see Table 3).

**Table 3:** Characteristics of included studies on the lockdown impact of older adult’s residents in nursing homes health

Authors, publication year, country	Aims of the study	Isolation measures	Data collection period	Study design		Residents (women)	Outcomes
Cortés-Zamora et al. 2020 [20]. Spain	To analyse psychological and functional consequences after the first wave of COVID-19 To analyze differences in psychological and functional variables, four phenotypes: COVID-19 no or low comorbidity” “COVID-19 no or high comorbidity” “COVID-19 yes/ low comorbidity” and “COVID-19 yes/high comorbidity	Not described	March-June 2020 June-November 2020	Longitudinal cohort study		215 (135 women)	At 3 months after the beginning of the COVID-19 pandemic: - Anxiety (HADS): prevalence increased 29.3% - Depression (HADS): prevalence increased 57.7% - Post-traumatic syndrome (TOP-8) present on 19.1%. of residents - Insomnia (IES) present on 93% of residents - Loss of functionality (FAC and MNA-SF) present in 47%
Crespo-Martín et al. 2022 [21] Spain	To understand underlying beliefs, values, and motivations behind individual health behaviors	Visitors ban Suspension of activities No interaction between residents (social distancing)	Two phases between May 2020 and December 2021	Interpretative exploratory (Sandelowski model)		24 (18 women)	Emotional impact (main topics): - Opposite feelings - Illness and death - Importance of routine - Feeling busy - Role of religious beliefs Support as a therapeutic instrument: - Family, roommates and staff
Curran et al. 2022 [22] Australia	To analyse changes in mental health (symptom burden) before the pandemic, following the first wave and following the second wave	Bespoke classification: mild RS: no non- essential visitors but alternative contact with family/carers/social network available (e.g. window visits or regular video calls) and at least 50% of usually scheduled internal activities continued; moderate RS: no or minimal alternative family/carers/social network visits and/or less than 50% of usually scheduled activities ongoing, but no restriction to individual rooms; severe RS: restriction to individual rooms for at least 3 weeks during wave two in addition to limited activities and social contact	October 1st, 2019, and February 1st, 2020 April 30th, 2020, and May 15th, 2020 September 27th, 2020, to October 18th, 2020	Longitudinal retrospective		91 (51 women)	- NPI-NH increased scores after the first wave (Mean: 19.0). Drop below initial scores after second wave (Mean: 15.5) - No significant changes comparing first and second wave scores with pre-pandemic
Egbujie et al. 2024 [23] Canada	Examine whether functional decline accelerated during the first wave of the COVID-19 pandemic (March to June 2020)	Restrictions on visits from family members or other visitors,	January 31, 2019, and June 30, 2020	Population-based longitudinal		199.598 (133.835 women)	- Higher rates of functional decline during the first wave of the COVD-19 pandemic: 23.3% vs 22.3%; (p < .0001) - Individuals 17% more likely to experiment functional decline during pandemic period - Environmental factors related to functional impairment: living in a large urban nursing home
El Haj et al. 2020 [24] France	To assess the increased symptoms of depression and anxiety in patients with Alzheimer’s disease during the pandemic	Significantly restricted visits since 15 March 2020 Activities considered as non-essential have been suspended, including restricting access to non-essential personnel	N/A	Quantitative, longitudinal		58 (37 women)	- Depression (HADS) Greater depression during confinement (Mean: 14.21; SD: 3.17) than PRE-COVID (Mean: 12.34; SD: 4.10) - Anxiety (HADS): Increased anxiety during confinement. (Mean: 13.24, SD: 3.39) than PRE-COVID (Mean = 11.38, SD: 4.36)
El Haj & Gallouj 2022 [25] France	To assess the loneliness level in patients with Alzheimer’s disease	Visitors ban Limited communication between residents Social distancing	N/A	Cross-section descriptive		63 (39 women)	- Depression (HADS) Greater depression during confinement (Mean: 14.21; SD: 3.17) than PRE-COVID (Mean: 12.34; SD: 4.10) - Anxiety (HADS): Increased anxiety during confinement. (Mean: 13.24, SD: 3.39) than PRE-COVID (Mean = 11.38, SD: 4.36)
Górski et al. 2021 [26] Poland	To assess the risk of suffering from depression and to explore the relationship between depression and cognitive impairment	Limitation of or ban on family visits, restricting contacts between the residents, and sometimes between the residents and staff. Ceased running group activities for the residents and holding communal meals	March- December, 2020	Longitudinal descriptive		273 (141 women)	- March: 16.1% MMSE scores within normal limits. No elevated risk of depression was detected - December: 10.3% MMSE scores within normal limits. Depression (DSI): 14.3% moderate risk: 14.3%, and high risk: 2.6%
Holston et al. 2024 [27] United States	Describe and understand the psychiatric symptoms experienced by older adults with mental health or neurocognitive conditions residing in skilled nursing facilities during the COVID-19 pandemic	Not described	March 2019 to March 2021	Descriptive		84 (49 women)	Cognitive impairment: - Pre COVID-19: Mean BIMS: 11.7 (moderate) - First year of COVID-19: Mean BIMS 11.6. (moderate) Depression levels: - Pre COVID-19: Mean PHQ-9 5.3 (mild) - First year of COVID-19: Mean PHQ-9 3.7 (none) First year of COVID: 67% symptoms related to altered behaviours, including delusions, disruptive physical and verbal behaviours, hallucinations, and inattentiveness
Huber & Seifert 2022 [28] Switzerland	To establish the correlation between loneliness and other independent variables	Not described	May 2021	Retrospective descriptive Multivariate regression analysis		828 (621 women)	Loneliness (LS): present in 23.5% Independent variables, subjective increase for: - Female sex - Less subjective enjoyment of life - Lower satisfaction with life - Less satisfaction with care received in residences
Johansson-Pajala et al. 2022 [29] Sweden	To understand the first wave impact of the pandemic (March–May 2020) on experiences of anxiety and loneliness	On April 1, 2020, visitor restrictions were implemented nationwide in nursing homes. In some nursing homes physical visits were replaced by social contact via telephone, video calls or through windows. However, home dwelling elders in Sweden were not subjected to visitor restrictions	March–May 2020	National retrospective cross-sectional		27,872 (18,871 women)	Ad hoc questionnaires: Anxiety: - Severe: 12% - Slight: 51% - No present: 37% Loneliness: - Often: 19% - Sometimes: 50% - No present: 30%
Kaelen et al. 2021 [30] Belgium	To explore what mental health and psychosocial needs identified and experienced by residents	Visitors ban Suspension of group activities No interaction between residents (social distancing) Outings ban	June 2020	Thematic content analysis		56 (35 women)	Main topics: - Loss of freedom - Loss of social life - Loss of distraction and stimulation - Loss of autonomy - Perception of wellbeing - Identified needs: social contact, freedom, activities, communication, and autonomy
Leontowitsch et al. 2023 [31] Germany	How older adults experienced lockdown from sociology, developmental psychology, and environmental gerontology perspectives	No visits from family members or friends No contact with other residents Little to no movement within the care home building Staff wearing full protective clothing Residents could only have one visit per week (starting from May 2020) for up to one hour by a close relative or friend, under strict conditions (mask-wearing, social distancing of 1.5 m, and no physical contact)	June 2020	Thematic content analysis		22 (14 women)	Three main themes emerged: [1] Care home settings and staff’s approach: - Ceasing or maintaining daily activities - Care home staff’s time and ability to turn a blind eye [2] Biographical sense of resilience: - Learning experiences from the past [3] A hierarchy of life issues: - Personal challenges - Challenges and hopes for society at large
Lood et al. 2021 [32] Sweden	To explore and describe the experiences of older adults	Visitors ban Outings ban Daily routines and essential activities could still be carried out Outside activities ban	June 2020	Triangulation through constant comparative analysis		10 (7 women)	Main topic: - It’s like living in a bubble Subtopics: - Live day to day without fear of the virus - Feel cared for - Having limited freedom - Missing out on the little extras
Nair et al. 2021 [33] Malaysia	To investigate the prevalence of depression, anxiety, and perceived social support	Not described	June -August 2020	Cross-sectional descriptive		224 (164 women)	- Depression (GDS-30): Prevalence 94.2% Severe depression: 50.9% - Anxiety (BAI): mild (36.6%) and moderate (38.4%) Perceived social support (MSPSS): low: 3.21 (SD: 0.89)
Pérez- Rodríguez et al. 2021 [34] Spain	To compare the functional, cognitive, and nutritional status before and after the first wave of the pandemic To assess differences between COVID and NON-COVID groups	Not described	May 21—June 21 2020	Observational multicentre		435 (341 women)	- Functional deterioration decreases of 20% in at least one BI category and 18.5% in at least one FAC category - Worsening of cognitive impairment of 22% in GDS and 25.9% in MEC Onset of depressive symptoms in 48% - Malnutrition prevalence (MNA-SF): increased by 36.8%. Weight loss: 38.4%
Rose et al. 2023 [35]	Examine the impact of the COVID-19 lockdown on anxiety symptoms	Restricted entry for visitors and nonessential personnel and ceased communal activities inside the facilities,	March 2019 and March 2021	Latent growth curve modelling with psychiatric diagnosis, psychiatric medication, and demographic factors included as covariates		1149 (806 women)	- Before the outbreak: Average anxiety level: 3.35 - After the outbreak: Average anxiety level: 3.41 - Mean difference (MD) in the intercepts (baseline anxiety levels): -0.058 (p < 0.005) - Effect size, measured by Cohen’s: d(-0.059), confidence interval ranging from -0.140 to 0.023 (suggests small effect)
Van der Roest et al. 2020 [36] Netherlands	To know the consequences of anti-COVID-19 measures on loneliness, mood, and behaviour problems	On March 20, Dutch government implemented a visitors ban in all nursing homes. Physical visits were replaced by social contact via telephone and video calls, or through windows. Many nursing homes closed social facilities and stopped daytime programs	May 2020	Cross-sectional descriptive		193 (N/A women)	Loneliness (MHI-5): 77% showed feelings of loneliness: - 50% moderately alone - 16% intensely - 11% very intensely alone

ACRONYMS:

BAI: Beck’s Anxiety Inventory

BI: Barthel Index

BIMS: the brief interview for mental status (BIMS)

BPRS: Brief Psychiatric Rating Scale

DSI: Depressive Symptom Inventory

FAC: Functional Ambulation Classification

GDS 30: Geriatric Depression Scale 30

GDS: Global Deterioration Scale

HADS: Hospital Anxiety and Depression Scale

interRAI ADL Hierarchy Scale: Hierarchy scale of Activities of Daily Living

IES: Insomnia in the Elderly Scale sub-scale A

LS: Loneliness Scale by Jong Gierveld, Van Tilburg and Dykstra (6 items)

MEC: Mini Cognitive Test (Spanish version for MMSE)

MHI-5: Mental Health Inventory 5-index

MMSE: Mini-Mental State Examination

MNA-SF: Mini Nutritional Assessment-Short Form

MSPSS: Multidimensional Scale of Perceived Social Support

NPI-NH: Neuropsychiatric Inventory Nursing Home

PHQ9: Patient Health Questionnaire-9

RS: Restriction Severity

TOP-8: Outcome Post-Traumatic Stress Scale

### Isolation measures

In twelve of the selected studies (66,66%), isolation measures aimed at preventing transmission and contagion among residents, with the visitor ban being the most implemented measure mentioned [21, 23, 25, 26, 29–32, 35, 36]. Curran et al. study used bespoke classification with varying degrees of visits restrictions [22]. Leontowitsch et al. reported on de-escalation of visits ban starting in May 2020 when residents were allowed one weekly visit for up to an hour by a close relative or friend, but only under strict conditions, including mask-wearing, social distancing of 1.5 m, and no physical contact [31]. Sometimes, visitor’s restrictions were replaced by regular telephone or video calls, or visits facilitated through windows [22, 29, 36].

Another commonly cited measure in the studies was social distancing between residents [21, 24–26, 30], and also between residents and staff [26]. Suspension of activities was also implemented [21, 22, 24, 26, 30, 31, 35, 36]. Finally, a common lockdown action was the outing ban [30, 32].

#### Functional state

Three of the 13 articles included (23.07%) revealing a decline in residents’ functional capacities at social health centers compared to pre-confinement levels.

Functional state was evaluated using various metrics, with the most common being the ability to perform basic activities of daily living (ADLs) using the Barthel Index (BI) [20, 34] and the Hierarchy Scale of Activities of Daily Living (interRAI ADL) [23] (See Table 4).

**Table 4:** Outcomes measured and assessment instruments

Outcome Measured	Instruments	Author, Year
*Functional state assessment*		
Activities of Daily Living (ADL)	Barthel Index (BI)	Cortés-Zamora et al. 2020 [20] Pérez-Rodríguez et al. 2021 [34]
	Global Deterioration Scale (GDS)	Pérez-Rodríguez et al. 2021 [34]
Ambulation	Functional Ambulation Classification (FAC)	Cortés-Zamora et al. 2020 [20] Pérez-Rodríguez et al. 2021 [34]
Nutritional condition	Mini Nutritional Assessment Short Form (MNA-SF)	Cortés-Zamora et al. 2020 [20] Pérez-Rodríguez et al. 2021 [34]
Frailty	Nursing Homes Frailty Scale (FRAIL)	Cortés-Zamora et al. 2020 [20]
Functional decline	Hierarchy scale of Activities of Daily Living (interRAI ADL)	Egbujie et al. 2024 [23]
*Cognitive state assessment*		
Cognitive status	Short Portable Mental Status Questionnaire Pfeiffer (SPMSQ)	Cortés-Zamora et al. 2020 [20] Pérez-Rodríguez et al. 2021 [34]
	Mini-Mental State Examination (MMSE)	Górski et al. 2021 [26]
	Mini-Cognitive Test (MEC) of Lobo	Pérez-Rodríguez et al. 2021 [34]
	The Brief Interview for Mental Status (BIMS)	Holston et al. 2024 [27]
*Psychological state and assessment instruments*		
Depression	5-item Geriatric Depression Scale of Yesavage (GDS-5)	Cortés-Zamora et al. 2020 [20]
	Hospital Anxiety and Depression Scale (HADS)	El Haj et al. 2020 [24]
	Depressive Symptom Inventory (DSI)	Górski et al. 2021 [26]
	Geriatric Depression Scale 30 (GDS-30)	Nair et al. 2021 [33]
	PHQ9: Patient Health Questionnaire-9	Holston et al. 2024 [27]
Anxiety	Hospital Anxiety and Depression Scale (HADS)	Cortés-Zamora et al. 2020 [20] El Haj et al. 2020 [24]
	National satisfaction survey: Are you affected by anxiety, restlessness or anguish?	Johansson-Pajala et al. 2022 [29]
	Beck’s Anxiety Inventory (BAI)	Nair et al. 2021 [33]
	Brief Psychiatric Rating Scale (BPRS)	Rose et al. 2023 [35]
Loneliness	Ad-hoc questionnaire: “During confinement I have felt: somewhat alone, very alone, not at all alone”	El Haj & Gallouj 2022[25]
	Loneliness Scale of Jong Gierveld (LS)	Huber & Seifert 2022 [28]
	National satisfaction survey: Do you feel lonely?	Johansson-Pajala et al. 2022 [29]
	Mental Health Inventory 5-index (MHI)	Van der Roest et al. 2020 [36]
Post Traumatic Stress Syndrome	Outcome Post-Traumatic Stress Scale (OPT-8)	Cortés-Zamora et al. 2020 [20]
Insomnia	Insomnia in the Elderly Scale (IES)	Cortés-Zamora et al. 2020 [20]
Neuro-psychiatric symptoms	Neuropsychiatric Inventory Nursing Home (NPI-NH)	Curran et al. 2022 [22]
Perceived Social Support	Multidimensional Scale of Perceived Social Support, (MSPSS)	Nair et al. 2021 [33]
Mood Statement	Have you often felt sad, discouraged, or hopeless? Have you felt less interest or pleasure in doing things?	Pérez-Rodríguez et al. 2021 [34]

In terms of functional decline, the study by Egbuije et al. found that individuals were 17% more likely to experience functional decline during the pandemic compared to the period before it [23]. In the study by Cortés-Zamora et al., 69.3% of nursing home residents were dependent on help for at least one ADLs at the beginning of the pandemic, reaching 77.2% at three months [20]. This study also reveals that 47% of the residents experienced persistent functional impairment three months into the pandemic, with a median functional loss of five points on the Barthel Index. Pérez-Rodríguez et al.’s study [34], 20.2% of residents declined by at least one dependency level category based on their BI. Older adults with moderate (BI 40–59) and severe dependence (BI 21–39) exhibited a more significant decline in ADL performance. Conversely, patients with high functionality (BI 90–100) experienced the least reduction in BI scores after three months of isolation measures [34]. Egbujie et al.’s study showed that residents in large homes and urban-located nursing homes were more likely to experience functional decline during the pandemic [23].

Regarding the ability to walk, in Pérez-Rodríguez et al.’s [34] study, 18.5% of participants experienced a decline in at least one category on the Functional Ambulation Classification Scale (FAC), with greater functional impairment observed in older adults with moderate to severe dependence. Conversely, those with higher pre-existing FAC scores (FAC = 5) exhibited the least decline [34].

In relation to nutritional state, a greater risk of malnutrition was observed among residents with COVID-19 at three months of follow-up compared to those without the disease [20]. Furthermore, 38.4% of residents experienced weight loss with a 36% increase in malnutrition compared to pre-pandemic levels [34].

#### Cognitive state

The studies that examined the impact at the cognitive and mental health level were 4, comprising 22.22% of the total, examined the impact on cognitive and mental health levels, as presented in Table 4.

Górski et al. and Holston et al. [26, 27] explored cognitive decline and its relationship with depression risk. Residents scoring below 24 in the MMSE decreased from 16.1% to 10.3% after strict isolation measures in December 2020. Additionally, 17% of residents showed a moderate to high risk of depression [26]; whilst Holston et al. reported very similar cognitive decline before and after one year of the pandemic onset and a reduction in severity depression symptoms [27]. Pérez-Rodríguez et al. [34] detected a decrease in the mean score of the MEC (Spanish version of the MMSE) from 24.5% before March 2020 to 22 after the first wave and the strict solation measures in June 2020, indicating mild cognitive impairment (scores from 24 to 20). Furthermore, using the Pfeiffer Short Portable Mental State Questionnaire (SPMSQ), Pérez-Rodríguez et al. noted a decline in scores for 14.1% of older adults [34]. This same tool was used by Cortés-Zamora et al. [20] but yielded no results in their study because of small sample size.

The results from the different studies suggest a decrease in MMSE scores during the first period of the pandemic (March to June 2020), with scores returning to baseline levels following the conclusion of the most restrictive social isolation measures.

#### Psycho-emotional state

The psycho-emotional health of the older adults during the initial months of confinement in nursing homes was evaluated in all the studies included in the review (100%). Most studies examined loneliness (*n* = 4), depression (*n* = 5) and anxiety (*n* = 5) (see Table 4, showing only quantitative studies). Other symptoms, including Post-Traumatic Stress Disorder (PTSD), insomnia and neuropsychiatric symptoms, were also evaluated. Studies assessing the psycho-emotional state used both quantitative and qualitative research designs.

### Psycho-emotional state collected in studies with quantitative design

Feelings described by residents were predominantly negative, reflecting participants’ altered perception of wellbeing. For instance, in Kaelen et al.´s study, participants manifested anger, stress, depressive feelings and an increase in the perception of discomfort, leading to a loss of hope and interest in life [30]. Fear and loneliness, caused by significant changes in participants’ occupations and routines as a result of COVID-19 restrictions, was also reported in other studies [21].

Concerning feelings of loneliness, Huber & Seifert [28] found that 23.5% rating their feelings of loneliness as low, with a mean score of 4.4. The relationship between loneliness and independent variables, including female sex, reduced life and dissatisfaction with the treatment received in the residence, is also evident. The remaining three studies assessing loneliness found its presence in 77% [36], 69% [29], and 81% [25] of their participants.

Regarding depression, Cortés-Zamora et al. [20] reported a 57.7% increase in the prevalence of clinically significant depressive symptoms three months into the pandemic. In contrast, Nair et al. [33] found that 94.2% of their study participants reported depressive symptoms, with 50.9% reporting severe depression. Górski et al. [26] found no participants at risk of developing depression in March 2020 but in December of that same year, 17% had a moderate to high risk. Finally, El Haj et al. [24] reported increased depression symptoms during confinement with a mean score of 14.21 compared to pre-COVID-19 means of 12.34. Holston et al.’s study reported that depression symptoms were resolved after the first year of the pandemic [27].

Anxiety was evaluated in four studiesusing different instruments (see Table 4), identifying the presence of this symptom in different proportions among the participants. Cortés-Zamora et al. [20] reported a 29.3% increase in anxiety symptoms prevalence three months after the onset of the pandemic and the implementation of isolation measures. El Haj et al. [24] observed mean scores during confinement of 13.24 compared to previous means of 11.38. Nair et al. [33] reported of mild anxiety in 36.6% of the subjects studied and moderate anxiety in 38.4%. Johanson Pajala et al. [29] found severe anxiety levels in 12% of participants and light in 51%. Finally, Rose et al. reported difference in the baseline anxiety levels between the pre-outbreak and post-outbreak periods: the average anxiety level increased from 3.35 to 3.41 [35]. Changes in routines could elevate stress levels among older adults, with 19.1% reporting symptoms of PTSD in Cortés-Zamora et al.’s study [20].

Insomnia, characterized by sleep disorders, was prevalent in 93.0% of participants in Cortés-Zamora et al. study [20]. This same study observed an increase in symptoms of post-traumatic stress disorder of 19.1%. Regarding perceived social support, studied by Nair et al. [33], the mean was 3.21 (SD = 0.89), indicating low levels. Curran et al. [22] evaluated neuropsychiatric symptoms (using the Neuropsychiatric Inventory Nursing Home [NPI-NH]) detecting an average increase up to 19 points (Inter Quartile Range, IQR, 8.0– 30.0) on the median (baseline median NPI- NH score = 17.0), after the first COVID-19 wave. A decrease below the initial scores after the second wave with averages of 15.5 points of the median (IQR: 7.0– 28.0), was detected.

Finally, Holston et al. found that 67% of individuals showed symptoms related to altered behaviours, such as delusions, disruptive behaviours (both physical and verbal), hallucinations, and inattentiveness after the first year of pandemic that were not perceived at pre-pandemic period [27].

### Psycho-emotional state collected in studies with qualitative design

In exploring the emotional experiences of older adults, different themes emerged in their narratives. The experiences of loss, concretized in the loss of freedom, the loss of social life (including contacts with family and friends), the loss of distraction and stimulation (such as missing the “little things in life”) and the loss of autonomy, were reported in the study by Kaelen et al. [30].

The residents expressed specific needs during this time, such as being in contact with other residents, need for family visits, need to be treated respectfully, need for activities and distractions, need for impartial information, and need for human contact [30]. Crespo-Martín et al. [21] also identified the importance of social support from family, roommates, and staff (as a therapeutic instrument), as well as the importance of routine and feeling busy, and the role of religious beliefs as strategies to overcome restrictions.

In conclusion, daily life in the nursing homes during the pandemic was similar to “living in a bubble” [32] wherein older adults experienced both a sense of protection and isolation.

In terms of what helped the most to older adults while lockdown Leontowitsch et al.’s study informed about the effects of nursing home settings and staff’s approach: phone calls from family and friends, the importance of maintaining daily routines and nursing home staff’s time for a daily chat was encountered helpful [31]. Ability to turn a blind eye from activities of an informal nature, officially not allowed, but tolerated by staff was also found supportive. Learning experiences of life from the past such as becoming a disciplined or patient person or surviving the perils of the Second World War gave older adults a biographical sense of resilience. A hierarchy of life issues: personal challenges but also challenges and hopes for society at large, were also useful for older adults to cope with the situation [31].

## Discussion

Given the study’s objective and findings, despite the known great impact of COVID-19 in social health centers, little has been investigated of lockdown measures on older adults residing in nursing homes. The results of this scoping review shed light on the effect of lockdown measures on older adult’s health and may guide the implementation of new actions aimed at preserving their wellbeing in case of future pandemics.

The 17 studies analyzed in this review employed diverse methodologies, included heterogeneous samples, and originated from various countries and geographical regions. Differences in centers and countries and variability in the implementation of isolation measures, were observed. Consequently, this variability may lead to contradictory results, hindering the extrapolation of data. However, when comparing the findings obtained in the different countries, no major differences or contrasts can be observed.

As shown by this review, the existing evidence, although limited, suggests that the isolation measures imposed in these centers had a negative impact at the functional, cognitive, and psycho-emotional level. Nonetheless, their potential unfavourable effects were predicted from the beginning. Thus, during the early phase of the first wave (March 21st, 2020) World Health Organization (WHO) stated that: “infection prevention and control (IPC) activities may affect the mental health and wellbeing of residents and staff, especially the use of PPE and restriction of visitors and group activities” [3].

Among these IPC activities, WHO recommended physical distancing in the facility: restrict the number of visitors (and secure screening for signs and symptoms of COVID infection), ensure physical distancing during group activities (or suspend them if not feasible); stagger meal times (if not possible, serve each resident a meal in their room); enforce a minimum of one meter distance between residents, and require residents and employees to avoid touching (e.g., shaking hands, hugging, or kissing). In the pandemic’s initial stages, older people living in nursing homes were recognized as a social group particularly susceptible to the impact of COVID-19 infection, with more severe involvement and higher mortality rate [37]. Therefore, it could be assumed that stricter lockdown measures were enforced in nursing homes compared to those for older adults residing in the community [28, 29].

Although some authors [21, 27, 29, 33, 34] did not mention lockdown measures in their study, it is assumed that the general actions launched by governments were established, such as: total isolation in single rooms (Malaysia) [38], measures allowing residents to see their families outside the nursing homes (Switzerland) [39] or “compassionate visits” in cases of severe or terminal illness (Spain) [40]. From the results provided by the studies it is not possible to extract a detailed analysis of whether the different isolation measures implemented differ in their negative impact on residents. However, it can be observed that in those studies reporting the application of a more stringent set of measures (i.e. visitors ban, suspension of activities and no interaction between residents), the findings suggest a negative impact at the emotional level with an increased level of anxiety [25, 36] or moderate risk of depression [25, 27] and offer narratives about feelings of loss of freedom, the sensation of living in a bubble or coping strategies [22, 32, 33].

### Decreased ability to perform basic activities of daily living

The functional impact of COVID-19 was observed among older adults in the worsening of the results measured by the BI. These aspects of ADLs indicated an increase in frailty in residents [20] point out the possible relationship between impaired walking and decreased walking speed, with the loss of muscle mass and strength caused by bedridden state or by physical immobility due to confinement. Moreover, the minimization of the direct contact of the caregiver with the residents to prevent contagion had a negative impact on their care, as shown by Cortés-Zamora et al. [20], who reported an increased risk of malnutrition, and by Pérez-Rodríguez et al. [34] who found an increase in malnutrition and weight loss in residents.

Older adults with greater independence in performing ADLs experienced less deterioration [20]. Cocuzzo et al. [41] remarked that the presence of multiple comorbidities in older adults who lacked sufficient attention suspended primary care appointments.

### Cognitive impairment and its relationship with social isolation

The cognitive impact was evident by a decline in the cognitive test score on different scales, which translated into increased deterioration among older adults [26, 34]. This deterioration may be related to prolonged immobility, lack of cognitive stimulation and limited physical exercise, and the appearance of symptoms of anxiety and depression [34].

The interventions of nurses and other health-care professionals were of great value in promoting the wellbeing of older adults, avoiding depression, and fostering their autonomy. However, it was even more crucial that older adults maintain frequent contact with their families, friends, and roommates. Before the COVID-19 pandemic, six categories of interventions were defined to prevent social isolation, loneliness and anxiety among older adults residing in nursing homes: social facilitators, psychological therapies, health and social care, interventions with animals and interventions of friendship and leisure [42, 43], all of which developed in the context of face-to-face and interpersonal contact. The relative studies indicated that one in five families in Canada spent at least 10 h a week caring for their relatives residing in social and health centers [44]. In Spain, more than half of the women who care for dependent people dedicated an average of 20 h a week in their care [45]. Other studies indicated that the most effective nonpharmacological interventions for people with dementia or cognitive disorders living in residential centers were individual visits from relatives or other close people and required two to three interactions per week for sustained benefits on mood to be observed [46]. Most of these interventions and the care provided by family members were interrupted throughout the confinement due to the COVID-19 pandemic. During public health like the COVID-19 pandemic, it may be necessary to adjust access to medical care to comply with required public health measures and restrictions. However, it is crucial to prioritize mental health care and wellbeing services for older adults in nursing homes as essential and uninterrupted [5].

### Worsening of neuropsychiatric symptoms

At the psycho-emotional level, the impact of this situation was observed in the increase in the prevalence of depression [20] or its risk [26] and the different degrees of loneliness [25, 29] reaching up to 77% of the study population [36]. These results underscore the negative impact that the pandemic and its precautionary measures have had on the mental health of residents and are in line with the results obtained in the review by Koszalinski et al. [15], who detected an increase in depression and anxiety during the pandemic compared to previous levels and an increase in neuropsychiatric symptoms, possibly caused by communication limitations. This same review points to the possible relationship between the interruption of the communication flow between caregivers and residents and a greater perception of social isolation, as well as a deep sense of loss. According to Górski et al. [26], there were no variations in the levels of depression symptoms between residents with and without neurocognitive impairment at the start of the visitation prohibition in long-term care facilities. But as time progressed the discrepancies stood out. It was noted that high-functioning people who could speak meaningfully and had visited their family frequently prior to the COVID-19 epidemic exhibited some of the typical symptoms of depression [26].

Another impact on a psycho-emotional level was the increase in the prevalence [33] and intensity of emotional anxiety [24]. This increase in anxiety can be explained by several reasons. First, people residing in nursing homes find in their residence a safe place to pursue their routines, which were abruptly interrupted during confinement [47]. Moreover, many of them were aware of their own vulnerability during that time and of the greater risk of a fatal outcome in case of contagion because they could see it happening to other residents. Finally, the increase in anxiety could be due to the reduction in physical contact with health care personnel [47]. The structural reorganization of residential centers, including the establishment of clean and contaminated areas and the creation of isolation and evacuation protocols or the detection of people with symptoms or a positive diagnosis of COVID-19 considerably disrupted resident’s routine [17].

The ADLs have a profound meaning for older adults living in nursing homes. Limiting nursing home residents’ choices of participating in activities, not considering the interests or abilities of residents when planning activities or introducing new routines (such as those imposed by lockdown), failed to meet the emergent needs of the residents, and increased their feelings of fragility and dependence [48]. This change in the routines of older adults could induce an increase in their stress levels, as detected in the study by Cortés-Zamora et al. [20].

### Feelings of loss and expressed needs

In the literature, emotional needs and deficiencies experienced during this time were detected along with a need to promote positive attitudes. Studies found a need for human contact, to be with other residents and with their families; the need to feel like a full-fledged person, to receive impartial information and respectful treatment; the need to promote their autonomy, to have space and time for recreation, activities and distractions [30]. To meet these needs, support has been identified in the literature that helps overcome confinement, such as social support from family, roommates, and staff (as a therapeutic instrument), the importance of routine and feeling busy, and the role of religious beliefs [21], as well as focusing on living day to day without fear of the virus and feeling cared for [32]. The identification of these needs can help the implementation of general measures in health centers and the promotion of personal strategies.

The social support perceived by older adults residing in nursing homes was found to be low during this period [33]. Experiences of loss of freedom, loss of social life, loss of distraction and stimulation, and loss of autonomy altered individuals’ perception of wellbeing, which resulted in a loss of hope and interest in life [30]. These factors may have affected individuals’ ability to adapt and overcome adverse life events such as the one experienced during isolation.

In the overall population of older people, there is a high correlation between the form and functionality of social networks and symptoms of anxiety and depression. By encouraging social network integration and involvement in community activities, public health programs might mitigate the perception of isolation and prevent the onset of depressive disorders [49]. Special attention must be paid in nursing home to avoid ageism: “a process of systematic stereotyping and discrimination against people based on age alone” [50]. Additionally, the oversimplification of the situation by designating older persons as a “highly vulnerable group” and neglecting the cultural, social, and contextual distinctions among them could have a substantial negative influence on the physical and psychological wellbeing of residents [51]. Considering that nursing home residents reported greater feelings of loneliness compared to older adults living at home, even before the COVID-19 pandemic [49], some of the consequences outlined in the present review might be predicted and mitigated.

### Implications of the findings

The results of this review can enhance preparedness and response to future pandemics and health crises, providing valuable insights to improve the planning and implementation of health policies and programs in nursing homes, ultimately increasing residents’ quality of life. Based on these e results, effective measures for future pandemics may include ensuring contact with family and friends, promoting physical activity within nursing homes (particularly when outing bans are installed), using public spaces like gardens and outdoor areas, facilitating communication of feelings and fears, and encouraging expression of religious feelings.

In the event of future pandemics, public authorities and stakeholders should install balanced measures that ensure the protection against infection in older adults living in nursing homes, but also provide emotional care and activities that protect them from increasing frailty. This review shows older adults ‘need for social support from family, roommates, and staff, which serves as a therapeutic instrument. Additionally, it highlights their need for recreation, promotion of positive attitudes, autonomy, and the protective role of religious beliefs in coping with isolation.

### Limitations

Our study has some limitations; a potential limitation is the variability in study designs, objectives, sample characteristics, and study contexts, despite efforts to identify evidence comprehensively through the search strategy and selection criteria. This variability complicates comparison or aggregation of results across studies. Nevertheless, the review offers a comprehensive mapping of existing evidence and its findings.

Another limitation of the study is the inclusion of cross-sectional studies, which precludes establishing causal relationships between isolation measures and their impact in different areas. It is challenging to discern whether results are attributable to isolation measures or other factors. Additionally, some of the data in the studies were collected retrospectively, introducing potential bias. Studies with lower levels of evidence were included to provide a breath of perspective and valuable insights into the research landscape on the impact of locking in the lives of older adults. Finally, although our scoping review had more inclusive criteria to capture a wide range of studies, we did not assess the quality of the original studies included in these reviews. Thus, caution is warranted when interpreting results.

Nonetheless, the synthesis highlights trends indicating the negative impact of isolation measures on residents’ health.

### Lines of investigation

How the COVID-19 precautionary measures affected older adults living in nursing homes is evident, but it is worth exploring how these measures might have affected differently other people of the same age group who spent months of confinement in their homes. Comparing the impact of confinement at a functional, cognitive, psychological, and emotional level between both population groups could be a future line of research. Additionally, exploring and comparing the resilience between the older adults and people of younger ages could be another line of research that will help to value and explore the adaptability of older adults and to combat ageism, which is very present in our society.

Another research direction could examine the specific isolation measures implemented in each nursing home and assess whether varying measures have different impacts. The reviewed articles discuss general measures at the country level rather than specific measures at individual nursing homes. Investigating the impact on infection rates based on the diverse isolation measures implemented in various nursing homes within each country could provide valuable insights.

## Conclusions

The analysis of the included articles indicated a broad impact of isolation measures on older adults residing in nursing homes, ranging from decreased in functional capacities compared to pre-confinement levels to increase in cognitive impairment during the first wave of the pandemic. The analyzed evidence describes the increased functional deterioration of residents, especially those with a moderate or severe level of dependency or in large homes and urban-located nursing homes, and the psycho-emotional impact with an increase in levels of anxiety or depression, and feelings of loss and loneliness during the months after the onset of the pandemic and the implementation of isolation measures. At the cognitive level, the results are more heterogeneous, and only some studies report greater deterioration after the first waves of the pandemic.

Based on these findings, there is urgency of developing new and more effective strategies to support the wellbeing of older adults in residential care settings. This may include innovative interventions aimed at addressing social isolation, facilitating meaningful social connections, promoting mental and emotional resilience, and ensuring comprehensive access to healthcare services. Additionally, there is a pressing need for more tailored and individualized care approaches that recognize and address the unique needs and vulnerabilities of older adults, particularly during times of crisis. By prioritizing the development and implementation of such approaches, we can better safeguard the health, dignity, and overall quality of life of the elderly population. If it becomes necessary to apply lockdown and isolation measures in nursing homes in the future, it is essential to translate the evidence analyzed in this review into clinical practice, so that strategies are implemented to promote the functional, cognitive, and emotional well-being of residents, mitigating the negative impact that these preventive measures have been shown to have.

## Data Availability

All data generated or analyzed during this study are included in this published article.

## References

[1] World Health Organization. Chronology of WHO response to COVID-19. 2021; Available from: https://www.who.int/es/news/item/29-06-2020-covidtimeline.

[2] Ramgobin D, Benson J, Kalayanamitra R, et al. The Economic Implications of COVID-19 in the United States. S D Med. 2020;73(5):218–22. 32579802

[3] Dyer AH, Fallon A, Noonan C, Dolphin H, O’Farrelly C, Bourke NM, O’Neill D, Kennelly SP. Managing the Impact of COVID-19 in Nursing Homes and Long-Term Care Facilities: An Update. J Am Med Dir Assoc. 2022;23(9):1590–602. 10.1016/j.jamda.2022.06.028. 35922016 10.1016/j.jamda.2022.06.028 PMC9250924

[4] Aalto UL, Pitkälä KH, Andersen-Ranberg K, et al. COVID-19 pandemic and mortality in nursing homes across USA and Europe up to October 2021. Eur Geriatr Med. 2022;13(3):705–9. 10.1007/s41999-022-00637-1. 35299261 10.1007/s41999-022-00637-1 PMC8929245

[5] American Geriatrics Society. American Geriatrics Society Policy Brief: COVID-19 and Nursing Homes. J Am Geriatr Soc. 2020;68(5):908–11. 10.1111/jgs.16477. 32267538 10.1111/jgs.16477 PMC7262210

[6] Eltaybani S, Suzuki H, Igarashi A, Sakka M, Amamiya Y, Yamamoto-Mitani N. Long-term care facilities’ response to the COVID-19 pandemic: A protocol of a cross-sectional, multi-site, international survey. Nurs Open. 2022;9(5):2506–17. 10.1002/nop2.1264. 35666062 10.1002/nop2.1264 PMC9348410

[7] Smith CB, Wong KLY, To-Miles F, et al. Exploring experiences of loneliness among Canadian long-term care residents during the COVID-19 pandemic: A qualitative study. Int J Older People Nurs. 2023;18(1): e12509. 10.1111/opn.12509. 36347829 10.1111/opn.12509 PMC9878008

[8] Merriam-Webster Dictionary. Available from: https://www.merriam-webster.com/dictionary/lockdown.

[9] Cornwell EY, Waite LJ. Social disconnectedness, perceived isolation, and health among older adults. J Health Soc Behav. 2009;50(1):31–48. 19413133 10.1177/002214650905000103 PMC2756979

[10] Tate DG, Kalpakjian CZ, Forchheimer MB. Quality of life issues in individuals with spinal cord injury. Arch Phys Med Rehabil. 2002;83(12 Suppl 2):S18–25. 10.1053/apmr.2002.36835. 12474168 10.1053/apmr.2002.36835

[11] American Psychological Association. Dictionary of Psychology. 2023. Available from: https://dictionary.apa.org/cognitive-functioning.

[12] World Health Organization. WHO fact sheet: Dementia. Available from: https://www.who.int/news-room/fact-sheets/detail/dementia.

[13] Keyes, Corey. Mental Health as a Complete State: How the Salutogenic Perspective Completes the Picture. Bridging Occupational, Organizational and Public Health: A Transdisciplinary Approach. 2013;179–192. 10.1007/978-94-007-5640-3-11.

[14] Verbiest MEA, Stoop A, Scheffelaar A, Janssen MM, van Boekel LC, Luijkx KG. Health impact of the first and second wave of COVID-19 and related restrictive measures among nursing home residents: a scoping review. BMC Health Serv Res. 2022;22(1):921. 10.1186/s12913-022-08186-w. Published 2022 Jul 15. 35841028 10.1186/s12913-022-08186-w PMC9286708

[15] Koszalinski RS, Olmos B. Communication challenges in social isolation, subjective cognitive decline, and mental health status in older adults: A scoping review (2019–2021). Perspect Psychiatr Care. 2022;58(4):2741–55. 10.1111/ppc.13115. 35582750 10.1111/ppc.13115

[16] Jackson N, Turner M, Paterson C. What are the holistic care impacts among individuals living through the COVID-19 pandemic in residential or community care settings? An integrative systematic review. Int J Older People Nurs. 2023;18(5): e12557. 10.1111/opn.12557. 37365716 10.1111/opn.12557

[17] Palacios-Ceña D, Fernández-Peña R, Ortega-López A, et al. Long-term care facilities and nursing homes during the first wave of the COVID-19 Pandemic: A scoping review of the perspectives of professionals, families and residents. Int J Environ Res Public Health. 2021;18(19):10099. 10.3390/ijerph181910099. 34639401 10.3390/ijerph181910099 PMC8508277

[18] Peters MDJ, Godfrey C, McInerney P, et al. Best practice guidance and reporting items for the development of scoping review protocols. JBI Evid Synth. 2022;20(4):953–68. 10.11124/JBIES-21-00242. 35102103 10.11124/JBIES-21-00242

[19] Tricco AC, Lillie E, Zarin W, et al. PRISMA Extension for Scoping Reviews (PRISMA-ScR): Checklist and Explanation. Ann Intern Med. 2018;169(7):467–73. 10.7326/M18-0850. 30178033 10.7326/M18-0850

[20] Cortés Zamora EB, Mas Romero M, TaberneroSahuquillo MT, et al. Psychological and Functional Impact of COVID-19 in Long-Term Care Facilities: The COVID-A Study. Am J Geriatr Psychiatry. 2022;30(4):431–43. 10.1016/j.jagp.2022.01.007. 35123862 10.1016/j.jagp.2022.01.007 PMC8782739

[21] Crespo-Martín A, Palacios-Ceña D, Huertas-Hoyas E, Güeita-Rodríguez J, Fernández-Gómez G, Pérez-Corrales J. Emotional Impact and Perception of Support in Nursing Home Residents during the COVID-19 Lockdown: A Qualitative Study. Int J Environ Res Public Health. 2022;19(23):15712. 10.3390/ijerph192315712. 36497786 10.3390/ijerph192315712 PMC9735792

[22] Curran E, Nalder L, Koye D, et al. COVID-19 and mental health: Impact on symptom burden in older people living with mental illness in residential aged care. Australas J Ageing. 2022;41(4):522–9. 10.1111/ajag.13042. 35129267 10.1111/ajag.13042 PMC9111336

[23] Egbujie BA, Turcotte LA, Heckman GA, Morris JN, Hirdes JP. Functional decline in long-term care homes in the first wave of the COVID-19 Pandemic: A population-based longitudinal study in five Canadian provinces. J Am Med Dir Assoc. 2024;25(2):282–9. 10.1016/j.jamda.2023.09.007. 37839468 10.1016/j.jamda.2023.09.007

[24] El Haj M, Altintas E, Chapelet G, Kapogiannis D, Gallouj K. High depression and anxiety in people with Alzheimer’s disease living in retirement homes during the covid-19 crisis. Psychiatry Res. 2020;291: 113294. 10.1016/j.psychres.2020.11329. 32763552 10.1016/j.psychres.2020.11329 PMC7357507

[25] El Haj M, Gallouj K. Loneliness of residents in retirement homes during the COVID-19 crisis. Encephale. 2022;48(4):477–9. 10.1016/j.encep.2021.05.001. 34238568 10.1016/j.encep.2021.05.001 PMC9310692

[26] Górski M, Buczkowska M, Grajek M, et al. Assessment of the Risk of Depression in Residents Staying at Long-Term Care Institutions in Poland During the COVID-19 Pandemic Depending on the Quality of Cognitive Functioning. Front Psychol. 2022;12:766675. 10.3389/fpsyg.2021.766675. Published 2022 Jan 3. 35046869 10.3389/fpsyg.2021.766675 PMC8761846

[27] Holston EC, Watts T, Yimmee S. Describing Psychiatric Symptoms Experienced by Older Adults in a Skilled Nursing Facility During the COVID-19 Pandemic. Issues Ment Health Nurs. Published online July 12, 2024. 10.1080/01612840.2024.2361329. 38995892

[28] Huber A, Seifert A. Retrospective feelings of loneliness during the COVID-19 pandemic among residents of long-term care facilities. Aging Health Res. 2022;2(1): 100053. 10.1016/j.ahr.2022.100053. 35018357 10.1016/j.ahr.2022.100053 PMC8739825

[29] Johansson-Pajala RM, Alam M, Gusdal A, et al. Anxiety and loneliness among older people living in residential care facilities or receiving home care services in Sweden during the COVID-19 pandemic: a national cross-sectional study. BMC Geriatr. 2022;22(1):927. 10.1186/s12877-022-03544-z. 36456904 10.1186/s12877-022-03544-z PMC9714409

[30] Kaelen S, Van den Boogaard W, Pellecchia U, Spiers S, de Cramer C, Demaegd G, et al. How to bring residents’ psychosocial wellbeing to the heart of the fight against Covid-19 in Belgian nursing homes-A qualitative study. PLoS ONE. 2021;16(3):e0249098. 10.1371/journal.pone.0249098. 33770110 10.1371/journal.pone.0249098 PMC7997017

[31] Leontowitsch M, Oswald F, Schall A, Pantel J. Doing Time: Experiences of Care Home Residents in Germany During the Early Phase of the COVID-19 Pandemic. Innov Aging. 2021;5(Suppl 1):529. 10.1093/geroni/igab046.2042. 10.1093/geroni/igab046.2042

[32] Lood Q, Haak M, Dahlin-Ivanoff S. Everyday life in a Swedish nursing home during the COVID-19 pandemic: a qualitative interview study with persons 85 to 100 years. BMJ Open. 2021;11(6): e048503. 10.1136/bmjopen-2020-048503. 34145018 10.1136/bmjopen-2020-048503 PMC8214988

[33] Nair P, Gill JS, Sulaiman AH, Koh OH, Francis B. Mental Health Correlates Among Older Persons Residing in Malaysian Nursing Homes During the COVID-19 Pandemic. Asia Pac J Public Health. 2021;33(8):940–4. 10.1177/10105395211032094. 34243684 10.1177/10105395211032094

[34] Pérez-Rodríguez P, Díaz de Bustamante M, Aparicio Mollá S, et al. Functional, cognitive, and nutritional decline in 435 elderly nursing home residents after the first wave of the COVID-19 Pandemic. Eur Geriatr Med. 2021;12(6):1137–45. 10.1007/s41999-021-00524-1. 34165775 10.1007/s41999-021-00524-1 PMC8222945

[35] Rose SG, Ward RN, Lind LM, Brown LM. Effects of the COVID-19 Pandemic on Anxiety Symptoms in Long-Term Care Residents: A Multilevel Growth Curve Analysis. J Am Med Dir Assoc. 2023;24(6):862–867.e1. 10.1016/j.jamda.2023.03.029. 37146644 10.1016/j.jamda.2023.03.029 PMC10067456

[36] Van der Roest HG, Prins M, van der Velden C, et al. The Impact of COVID-19 Measures on Well-Being of Older Long-Term Care Facility Residents in the Netherlands. J Am Med Dir Assoc. 2020;21(11):1569–70. 10.1016/j.jamda.2020.09.007. 33036911 10.1016/j.jamda.2020.09.007 PMC7833500

[37] World Health Organization. Preventing and managing COVID-19 across long-term care services: Policy brief. 2020; Available from: https://www.who.int/publications/i/item/WHO-2019-nCoV-Policy_Brief-Long-term_Care-2020.1.

[38] Hasmuk K, M Sallehuddin H, Tan M, Cheah K, Ibrahim R, Chai ST. The Long Term Care COVID-19 Situation in Malaysia. The Long-Term Care Policy Network. 2020; Available from: https://ltccovid.org/wp-content/uploads/2020/05/Malaysia-LTC-COVID-situation-report-30-May.pdf.

[39] Chimento-Díaz S, Espino-Tato I, Garcia-Alonso JM, Cantero-Garlito PA. Lessons Learned: Occupational Therapy in Nursing Homes during the First Wave of COVID-19 in Spain. Healthcare (Basel). 2022;10(1):117. 10.3390/healthcare10010117. 35052281 10.3390/healthcare10010117 PMC8775346

[40] Zalakain, J. Davey, V. & Suárez-González, A. The impact of COVID-19 on users of Long-Term Care services in Spain. Long-Term Care Policy Network. 2020; Available from: https://ltccovid.org/wp-content/uploads/2020/05/LTCcovid-Spain-country-report-28-May-1.pdf.

[41] Cocuzzo B, Wrench A, O’Malley C. Balancing Protection from COVID-19 and the Need for Human Touch in Nursing Homes. J Am Geriatr Soc. 2020;68(12):2749–51. 10.1111/jgs.16861. 32949146 10.1111/jgs.16861 PMC7646659

[42] Beogo I, Tchouaket EN, Sia D, et al. Promising best practices implemented in long-term care homes during COVID-19 pandemic to address social isolation and loneliness: a scoping review protocol. BMJ Open. 2022;12(1): e053894. 10.1136/bmjopen-2021-0538941. 34980621 10.1136/bmjopen-2021-0538941 PMC8724591

[43] Gardiner C, Laud P, Heaton T, Gott M. What is the prevalence of loneliness amongst older people living in residential and nursing care homes? A systematic review and meta-analysis. Age Ageing. 2020;49(5):748–57. 10.1093/ageing/afaa049. 32396600 10.1093/ageing/afaa049

[44] Turcotte M, Sawaya C. Senior care: Differences by type of housing. Stat Can [Internet]. 2015; Available from: https://www150.statcan.gc.ca/n1/pub/75–006-x/2015001/article/14142-eng.pdf.

[45] National Statistical Institute. Proyecciones de Población [Internet]. 2018; Available from: https://www.ine.es/prensa/pp_2018_2068.pdf.

[46] Seitz DP, Brisbin S, Herrmann N, et al. Efficacy and feasibility of nonpharmacological interventions for neuropsychiatric symptoms of dementia in long term care: a systematic review. J Am Med Dir Assoc. 2012;13(6):503–506.e2. 10.1016/j.jamda.2011.12.059. 22342481 10.1016/j.jamda.2011.12.059

[47] Mo S, Shi J. The Psychological Consequences of the COVID-19 on Residents and Staff in Nursing Homes. Work Aging Retire. 2020;6(4):254–9. 10.1093/workar/waaa021. 34192005 10.1093/workar/waaa021 PMC7665707

[48] Mondaca M, Josephsson S, Katz A, Rosenberg L. Influencing everyday activities in a nursing home setting: A call for ethical and responsive engagement. Nurs Inq. 2018;25(2): e12217. 10.1111/nin.12217. 28762593 10.1111/nin.12217 PMC6084291

[49] Santini ZI, Jose PE, York Cornwell E, et al. Social disconnectedness, perceived isolation, and symptoms of depression and anxiety among older Americans (NSHAP): a longitudinal mediation analysis. Lancet Public Health. 2020;5(1):e62–70. 10.1016/S2468-2667(19)30230-0. 31910981 10.1016/S2468-2667(19)30230-0

[50] García-Soler Á, Castejón P, Marsillas S, Thompson L, Díaz-veiga P. Ageism and COVID-19 : study of social inequality through opinions and attitudes about older people in the coronavirus crisis in Spain. Long-Term Care Policy Network. 2020;1–13. Available from: https://ltccovid.org/wp-content/uploads/2020/08/COVID-and-ageism-an-attitudes-survey-in-Spain.pdf.

[51] Vale MT, Stanley JT, Houston ML, Villalba AA, Turner JR. Ageism and Behavior Change During a Health Pandemic: A Preregistered Study. Front Psychol. 2020;11: 587911. 10.3389/fpsyg.2020.587911. 33329247 10.3389/fpsyg.2020.587911 PMC7710520

